# Biological and environmental correlates of cougar (*Puma concolor*) survival in west central Alberta

**DOI:** 10.1371/journal.pone.0357879

**Published:** 2026-10-28

**Authors:** Carter J. Littlefair, Paul F. Frame, Mark A. Edwards, Andrew E. Derocher

**Affiliations:** 1 Department of Biological Sciences, University of Alberta, Edmonton, Alberta, Canada; 2 Fish and Wildlife Stewardship, Alberta Environment and Protected Areas, Edmonton, Alberta, Canada; 3 Office of the Chief Scientist, Alberta Environment and Protected Areas, Edmonton, Alberta, Canada; 4 Department of Renewable Resources, University of Alberta, Edmonton, Alberta, Canada; Alaska Pacific University, UNITED STATES OF AMERICA

## Abstract

Managing large carnivores benefits from understanding their vital rates, including survival. For cougars (*Puma concolor*), the hazards relevant to survival across the landscape are poorly understood. The risks that landscape features present can change temporally, increasing survival in one season while creating mortality risk in another. Between 2016 and 2021, 80 cougars were radio-collared and tracked in west central Alberta, Canada, for a total of 1082 cougar months with a mean monitoring time of 13.5 months/individual. We documented 37 deaths, determined causes of mortality, generated survival estimates using Kaplan-Meier models by sex and age class annually and for two seasons (i.e., warm and cold), and investigated factors affecting survival using Cox proportional hazard models. Humans were responsible for 29 of the 37 documented mortalities, including harvest, removal of problem individuals, landowner removals, incidental trapping, and vehicle collisions, with harvest representing the largest single source of mortality (16/37 deaths) for both sexes. However, we were unable to detect a difference in survival estimates between sexes or age classes and found no evidence of a male-biased harvest. Mean annual survival was 0.60 (95% CI: 0.50–0.72) and varied seasonally with lower survival in the cold season (0.62, 95% CI: 0.52–0.74) than the warm season (0.96, 95% CI: 0.92–1.00). Using Cox proportional hazards models (Andersen-Gill), model selection identified a best-supported mixed model combining biological, environmental, and anthropogenic features: season, proximity to residences, proximity to secondary roads, linear-feature crossing rate, and step length.

## Introduction

Large apex carnivores play important roles in the function of ecosystems and affect herbivore populations, which may result in cascading effects that alter vegetation, avifauna, and mesocarnivore ecology through intraguild competition [1–4]. Within North America, land conversion, hunting, and directed persecution (e.g., bounties) reduced the abundance of large carnivores by the early twentieth century [5,6]. Since then, effort has been made to restore many of these populations. Effectively managing large carnivores, and ensuring population viability, is improved with an understanding of carnivore population dynamics, and thus vital rates such as survival [7,8].

Carnivore survival is influenced by a suite of biological, environmental, and anthropogenic factors. Biological traits such as sex, age, and movement behaviour can influence exposure to risk [9–12]. Environmental conditions, including habitat type, weather, and terrain, influence where and how cougars move across the landscape and the availability of prey, thereby affecting mortality risk [11,13–15]. Anthropogenic features such as road networks, settlements, and human access to cougar habitat (roads, trails, etc.) often have the strongest influence, with human-caused mortality exceeding natural causes in many systems (e.g., conflicts and harvest) [16–19].

Cougars (*Puma concolor*) are an apex predator with a wide distribution across North and South America and their populations are often regulated through hunting (e.g., harvest quotas) and other mortality from anthropogenic sources (e.g., conflicts) that are often higher than natural factors [16–19]. Natural sources of mortality include intraspecific aggression [18], disease [17], infanticide [20], and predation from both conspecifics and other species [21]. While humans may be the main mortality factor, cougar survival rates are influenced by both biological and environmental factors [7,16]. Biological factors affecting cougar survival include age, sex, presence and number of offspring, and behaviour (e.g., home range fidelity, linear feature crossing rate) [22–24]. Age can influence harvest risk because larger individuals are preferred, but also through use of the environment [25]. Subadults and adults differ in their space use and habitat use with subadults often leaving natal ranges and dispersing into new areas to establish home ranges away from adults and siblings [26,27]. During dispersal, mortality risk increases from intraspecific competition or anthropogenic sources due to naïve behaviours in novel habitats [28]. Resident adults with established home ranges may also differ in risk, with individuals exhibiting high home range fidelity potentially experiencing higher survival due to spatial familiarity and increased efficiency in acquiring prey compared to individuals with lower fidelity [29]. Further, dependent offspring can influence survival of their mothers through intraspecific conflict with infanticidal males, or through risks involved with occupying lower quality habitats to avoid males [11].

Environmental factors affecting cougar survival include weather, landcover types, topography, and anthropogenic features [30,31]. Habitat characteristics can influence survival through effects on prey availability, shelter, nursery-site availability, and exposure to human-caused mortality [22]. Weather may also affect mortality risk by altering hunter success, such as improving track detection following snowfall. Different habitat types can present distinct risks and benefits; for example, edge habitats may facilitate hunting opportunities, whereas densely forested areas may provide cover for nursery sites [32–34]. While studies have examined the role of environmental variables on cougar habitat selection, the influence of habitat on survival is poorly understood.

Anthropogenic features can affect cougar survival both directly and indirectly. Roads, residences, and other human developments can increase mortality risk by improving hunter access, increasing the likelihood of conflict, or facilitating landowner removals [21]. At the same time, cougars often modify their behaviour in response to human activity, including shifting activity patterns and avoiding heavily used areas, which can reduce revisitation rates of cached prey, which can lead to higher kill rates and greater energy expenditure [31]. Cougars show flexible behaviour by adjusting activity times in response to anthropogenic development and use areas with human presence at less busy times [22,35]. While cougars generally avoid areas with high human densities, this avoidance itself may reduce survival if they select lower quality habitats to avoid humans [30,36,37]. Individual landscape features may simultaneously provide benefits and risks, predicting their net effect on survival is challenging. For example, secondary roads may improve travel efficiency while also increasing vulnerability to harvest by making tracks easier for hunters to locate [30].

Understanding how biological, environmental, and anthropogenic factors influence cougar survival is essential for informing management strategies, particularly in systems where regulated harvest is a primary source of mortality. Identifying which landscape features are associated with increased mortality risk can help clarify how human access and development shape harvest exposure and contribute to more spatially explicit conservation and management planning.

The objective of this study was to assess causes of mortality, estimate survival rates of cougars, and examine how biological, environmental, and anthropogenic factors influence mortality risk. We used satellite telemetry data collected from 2016 to 2021 in west-central Alberta, Canada, incorporating known mortality locations. Because the region is managed for sport hunting, we expected harvest to be the primary source of mortality. We tested four hypotheses regarding the factors influencing cougar survival. We hypothesized that (1) biological and behavioural factors, including age, sex, and home range fidelity affect survival; (2) environmental conditions, including season and habitat, influence survival by altering risk exposure; (3) mortality risk increases with anthropogenic infrastructure, including roads and residences, where human activity and hunting pressure would be higher; and (4) the best-supported model would include variables from all three categories, reflecting the combined influence of biological, environmental, and human-driven processes on survival.

## Study area

The study area was in west central Alberta, Canada, south of Whitecourt, west of Rocky Mountain House, north of Sundre, and east of the Rocky Mountains (Fig 1). Alberta uses 32 cougar management areas (CMAs) and sex selective harvest quotas for each CMA [38]. Harvest limits are established for individual CMAs and, since 2019, have generally been set at approximately 14–16% of the estimated cougar population within each CMA. Provincial compulsory registration data indicates that human-caused mortality has declined since peaking in 2015. Cougars were collared in 3 CMAs, and we defined the study area as the 100% minimum convex polygon (Minimum Bounding Geometry tool in ArcGIS, version 10.7.1, Environmental Systems Research Institute, Redlands, CA) encompassing all telemetry locations, which included additional CMAs that cougars dispersed into. These CMAs were selected because they capture the broad gradient of ecological conditions and human influence experienced by cougars in Alberta, ranging from the eastern edge of cougar distribution to predominantly Crown wildlands in the west. The area transitions from mountainous terrain to prairies and agriculture land, and contains alpine, subalpine, upper and lower foothills, and mixed forest ecoregions [39]. Forested areas were comprised largely of white spruce (*Picea glauca*), black spruce (*P. mariana*), lodgepole pine (*Pinus contorta*), jack pine (*P. banksiana*), and tamarack (*Larix laricina*) with patches of trembling aspen (*Populus tremuloides*) and balsam poplar (*P. balsamifera*) (Downing and Pettapiece, #75) [39]. The main prey in the area was white-tailed deer (*Odocoileus virginianus*) with other common ungulate and non-ungulate prey including mule deer (*O. hemionus*), moose (*Alces alces*), elk (*Cervus elaphus*), and beaver (*Castor canadensis*) [40]. Other large carnivores included wolves (*Canis lupus*), black bears (*Ursus americanus*), and grizzly bears (*U. arctos*). Logging and oil and gas activity is common throughout the landscape and have resulted in extensive secondary road networks.

**Fig 1:**
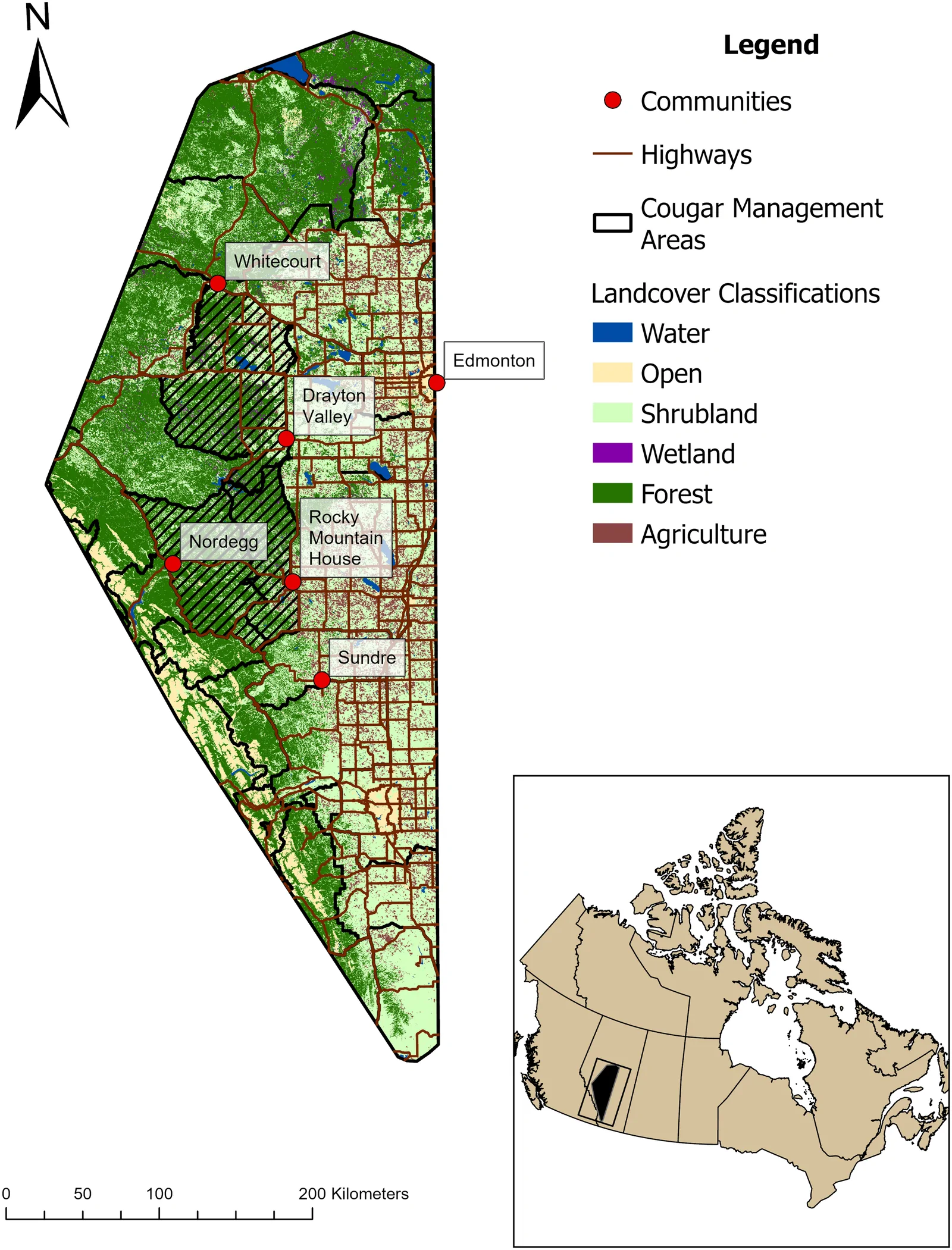
Study area in west central Alberta, Canada, showing the cougar management areas (CMA), hatched areas represent CMAs where collars were deployed. Insert shows the study area designated as the 100% minimum convex polygon encompassing all collar points. CMA boundaries were obtained from Alberta Environment and Protected Areas [38].

## Methods

Cougars were located by searching roads for tracks and hounds were used to trail and tree cougars that were then immobilized using a Dan-inject CO2 rifle (Dan-Inject, Kolding, Denmark) and darts containing medetomidine (0.05–0.075 mg/kg) and Telazol (1.7–2.6 mg/kg) (MZT) [29,41]. MZT was reversed following handling using atipamezole (0.4 mg/kg). Immobilized animals were removed from trees, weighed, sexed, aged using gum-line recession, tooth wear, and color and spotting of pelage [18], and fitted with a geographic positioning system (GPS) collar (Vectronic Aerospace Gmbh, Berlin, Germany) linked to satellites (Iridium Communications, McLean, VA). All capture, handling, and monitoring procedures were conducted under a Research Permit issued by the Alberta Environment and Parks, Resource Stewardship Division, Fish and Wildlife Stewardship Branch (Research Permit No. GP 58627). The permit authorized cougar capture, immobilization, collaring, biological sample collection, telemetry, and associated field activities conducted as part of the Alberta Cougar Adaptive Management Project. This research was carried out in accordance with the guidelines of the Canadian Council on Animal Care (CCAC 2003) and approved under the Alberta Wildlife Animal Care Committee Class Protocol #12 (Government of Alberta Research Permit #58627). Given the high density of roads, we believe that our sample is representative of cougars in the area. Age classes were: subadult (18–24 months), and adults (> 24 months) [21]. GPS locations were recorded every 7 hours and we used a t-test to compare inter-sex monitoring duration. We used location data to locate nursery sites and visited them to determine litter size.

### Survival and hazard analysis

Survival estimates were determined annually and seasonally using Kaplan-Meier models [4]. To assess seasonal variation in survival we defined two seasons: cold (November – March) and warm (April–October) [23,42]. Hunting season began December 1 in all CMAs and closed at the end of February or when quotas were filled. Alberta also has an autumn cougar hunting season in certain Wildlife Management Units that prohibits the use of dogs from 1 September to 31 March, however no cougars were harvested in our study area during this time.

Individual covariates included age class and sex, with adult females further divided into females with and without dependent offspring. Litter size was recorded by counting the number of kittens at nursery sites visits of individuals suspected of having offspring based on movement data. Differences in overall and cause-specific mortality between sexes (i.e., overall mortality, harvest, natural, and collision deaths) were evaluated using exact logistic regression (ELR) with the elrm package in R [43].

Hazards were examined using a series of univariate Cox proportional hazard models with the Anderson-Gill model for staggered entry in the program R [7,44,45]. No random effects were included in the Cox proportional hazards models, as each individual cougar represented an independent sampling unit and repeated measures were accounted for through the Anderson-Gill formulation for staggered entry. Hazard ratios near 1 were not considered biologically insightful as they signified a minute change in hazard.

Landcover was determined at each cougar location using the Government of Alberta landcover dataset [46]. Residential building density was determined within a 1 km radius of each cougar location. Residential density (annual and seasonal) were used as covariates [47]. Euclidean distances to secondary roads and residential buildings were determined for each cougar location using the Near tool in ArcGIS. Closer locations were expected to have more of an effect than those further away so we created exponential decay layers (e^(-d/a)^), where *d* was the distance to the feature and *a* was the buffer value [23,48]. This functional form is commonly used to model distance-dependent ecological effects where influence is expected to decrease continuously rather than abruptly. To reduce model complexity we used a subset of variable distances used by Smereka, Frame (23), and created layers with buffer radii of 50 m, 150 m, and 250 m for secondary roads and residential buildings. A large range of buffer layers was examined to account for cougar response to anthropogenic features as well as human access to these areas [49]. Buffer layers were created, and Euclidean distances were measured using the Buffer and Near tool in ArcGIS. Each feature’s buffer layer was ranked using Cox proportional hazard models as an annual hazard and the lowest Akaike’s information criterion adjusted for small sample size (AIC_c)_ for each layer was then used in each seasonal analysis [22].

Variables with multiple buffer layers examined were incorporated into candidate models and the model for each variable with the lowest AIC_c_ was used in each season [50]. Preliminary exploration removed any variables that showed no significance when tested with a univariate cox proportional hazard model (S2 File).

Step length (i.e., distance between consecutive locations) and crossing rates of linear features (i.e., powerlines, pipelines, cutlines, seismic lines, and railways from [51–53] and secondary roads were examined to calculate mortality risk. Secondary roads (i.e., all unpaved roads) crossing rate was examined separately from the linear features. All roads, primary and secondary, were separated from other linear features as the potential hazards and use of roads may differ from other linear features. Linear features and roads were mapped in ArcGIS, and crossings were identified by intersecting straight-line movement segments between successive GPS locations with mapped features. The individual crossing rate was determined as crossings/step.

Home range fidelity was treated as a binary variable, within vs. outside the previous year’s 95% kernel density home range. For each individual where a mortality event occurred, the previous year’s home range was determined using the 95% fixed kernel density in R [21,54].

Variables were grouped into three categories—biological, environmental, and anthropogenic—and the top model within each category was determined using AIC_c_. The top model from each category were then combined to determine which variables were retained in the overall best-supported mixed model. Prior to model selection, we assessed collinearity among anthropogenic covariates using Pearson correlation coefficients and variance inflation factors (VIF). Variables were considered potentially collinear when r > 0.70 or VIF > 3 [55,56].

Mortality sites were investigated within 3 days of receiving a notification from the collars. The site was investigated for evidence to help understand cause of death (tracks, scats, signs of struggle, etc.) Field necropsies were conducted to look for subcutaneous hemorrhaging and punctures in the hide consistent with predator attacks.

## Results

Between 2016 and 2021, 80 cougars were collared (56 females, 24 males) and monitored for a total of 1082 cougar-months (Table S1 in S1 File). Mean monitoring duration was 13.5 ± 1.2 months (range = 0.4–48.2) and did not differ significantly between sexes (t = 1.99, P = 0.16, df = 78; males: 11.2 ± 1.6 months, 0.6–35.1; females: 14.5 ± 1.6 months, 0.4–48.2). Of the 80 monitored cougars, 37 died. Harvest (43.2%; 16/37) and incidental trapping (21.6%; 8/37) were the primary mortality sources (Table 1). Human-caused mortality observed within our study areas followed trends similar to those observed provincially, suggesting that mortality patterns documented in this study are representative of broader CMAs in Alberta (Government of Alberta, unpublished data).

**Table 1:** Sex-specific causes of mortality for cougars monitored using satellite telemetry in west central Alberta, Canada, from 2016 to 2021.

	Hunter Harvest	Problem Removals	Landowner Removals	Incidental Trapping	Roadkill	Natural	Unknown	Total
Female	9 (16%)	1 (2%)	1 (2%)	5 (9%)	2 (4%)	7 (13%)	1 (2%)	26 (46%)
Male	7 (29%)	1 (4%)	0	3 (13%)	0	0	0	11 (46%)

“Problem Removals” refers to animals removed due to human-wildlife conflict (e.g., livestock depredation or perceived threat to human safety); “Landowner Removals” refers to animals killed by private landowners under authorized or permitted control actions; “Incidental Trapping” refers to unintentional mortality in traps set for other target species.

Eighteen litters were identified from collared females. Survival did not differ between females with and without offspring (Fig S1 in S1 File), so females were pooled. Kaplan-Meier survival models found overlapping 95% confidence intervals between adult and subadult males and females both annually and seasonally (Table S2 in S1 File). We found no significant difference between these sex and age classes, although mortality was numerically higher for both sexes during the cold season (Table 2; Table S2 in S1 File).

**Table 2:** Number of cougar mortalities by sex, age class, and season among individuals monitored using satellite telemetry in west central Alberta, Canada, from 2016 to 2021. Seasons were classified as warm (April-October) and cold (November-March).

Sex	Age Class	Season	Deaths
Female	Adult	Cold	20
Male	Adult	Cold	10
Female	Adult	Warm	4
Female	Subadult	Cold	2
Male	Subadult	Cold	1

We found no significant difference in overall mortality risk between sexes (β = −0.02, P = 1.00). Harvest-specific survival did not differ between males and females (β = 0.76, P = 0.23). Natural mortalities, including predation, disease, and starvation, occurred only among females, but were not significantly different between sexes (β = −1.57, P = 0.09). Wolves killed three adult females, two of which had dependent offspring. Two additional females died from vehicle collisions, with no sex difference in this mortality source (β = −0.13, P = 1.0).

Given that sex and age class had no significant difference on survival estimates or mortality causes, all individuals were pooled for subsequent analyses. Pooled annual survival probability was 0.60 (95% CI = 0.50–0.72). Seasonal survival estimates indicated a significantly lower cold season survival rate (0.62, 95% CI = 0.52–0.74) compared to the warm season (0.96, 95% CI = 0.92–1.00).

We determined the previous year’s home range of 15 individuals that died, 3 of which died outside of the previous year’s home range while the other 12 died within it. Fidelity to home range did not appear to influence the mortality risk.

Two biological, seven environmental, and six anthropogenic variables were excluded due to a lack of significance in univariate testing (Tables S3–S5 in S1 File). Univariate analyses identified one biological and one environmental variable that were significantly associated with mortality risk: step length (HR = 1.12, P = 0.053, LR = 0.002) and season (HR = 12.04, P = < 0.001, LR = 0.000005) (LR = Likelihood ratio test) (Table 3).

**Table 3:** Results of univariate Cox proportional hazard models evaluating the influence of biological, environmental, and anthropogenic variables on cougar survival in west-central Alberta, Canada, from 2016 to 2021.

Category	Variable	Hazard Ratio	P	Likelihood Ratio Test P
Biological	Step Length	1.12	0.053	1.59 x 10^−3^
Environmental	Season	12.04	< 0.001	< 0.001
				< 0.001
Anthropogenic	Residence Density	1.16	< 0.001	< 0.001
	Residential Distance 150m	18.52	< 0.001	< 0.001
	Secondary Road Distance 50m	4.80	< 0.001	< 0.001
	Secondary Road Crossing	4.56	< 0.001	< 0.001
	Linear Feature Crossing	1.26	< 0.001	< 0.001

Pairwise correlations among anthropogenic covariates were generally low to moderate (maximum |r| = 0.67; Table S6 in S1 File). All variance inflation factors were below the threshold of concern (VIF < 3), ranging from 1.04 to 1.89. For the anthropogenic variables, a 150 m buffer to residential sites (AIC_c_ = 352.68) and 50 m to secondary roads performed best (AIC_c_ = 359.04) (Table 4). Five anthropogenic variables showed significant relationships with cougar survival: residential density (HR = 1.16, P = < 0.001, LR = 0.0005); distance to residence with a 150 m decay buffer (HR = 18.52, P = < 0.001, LR = 0.00001); distance to secondary road with a 50 m decay buffer (HR = 4.80, P = < 0.001, LR = 0.0004); secondary road crossing rate (HR = 4.56, P = < 0.001, LR = 0.0003); and linear feature crossing rate (HR = 1.26, P = < 0.001, LR = 0.0002) (Table 3). All combinations of anthropogenic variables were examined, and the best fit model was distance to secondary roads + distance to residential areas + linear feature crossing rate (AIC_c_ = 338.31) (Table 5).

**Table 4:** Akaike Information Criterion corrected for small sample (AIC_c_) size ranking of covariates to be used within the Cox proportional hazards models for cougars within Alberta, Canada, from 2016 to 2021.

Classification	AIC_c_	Delta AIC_c_	AIC_c_ Wt.	LL	K
Residential (150 m)	352.79	0.00	0.55	−175.34	1
Residential (50 m)	353.95	1.16	0.31	−175.92	1
Residential (250 m)	355.56	2.77	0.14	−176.73	1
Secondary Road (50 m)	359.15	0.00	0.78	−178.52	1
Secondary Road (150 m)	362.42	3.27	0.15	−180.16	1
Secondary Road (250 m)	363.95	4.81	0.07	−180.93	1

**Table 5:** Competing anthropogenic models ranked by AIC_c_ based on Cox proportional hazard analyses of cougar survival in west-central Alberta, Canada, from 2016 to 2021.

Combination	AIC_c_	Delta AIC_c_	AIC_c_ Wt.	LL
Distance to secondary road (50 m) + Distance to residence (150 m) + Linear feature crossing rate	338.31	0.00	0.21	−166.16
Residential density + Distance to secondary road (50 m) + Distance to residence (150 m) + Linear feature crossing rate	338.69	0.38	0.17	−165.34
Distance to secondary road (50 m) + Distance to residence (150 m) + Linear feature crossing rate + Secondary road crossing rate	339.44	1.13	0.12	−165.72
Distance to secondary road (50 m) + Distance to residence (150 m) + Secondary road crossing rate	339.71	1.40	0.10	−166.86
All 5 variables	340.01	1.70	0.09	−165.00

The final mixed model analysis included all variables from the top-performing model within each category. We examined all possible combinations of step length, season, distance to secondary roads (50 m), distance to residences (150 m), and linear feature crossing rate. The top-supported model included all five variables (AIC_c_ = 317.32; Table 6). In the final model linear feature crossing rate, season, and distance to residence (150 m) (all P < 0.001), and distance to secondary road (P = 0.023) were significant predictors, whereas step length was not (P = 0.26) (LR = 4E-12, Table 7).

**Table 6:** Top five multivariate Cox proportional hazard models ranked by AIC_c_ for cougar survival in west-central Alberta, Canada, from 2016 to 2021. Models include combinations of biological, environmental, and anthropogenic variables.

Combination	AIC_c_	Delta AIC_c_	AIC_c_ Wt.	LL
Step Length + Season + Distance to secondary road (50 m) + Distance to residence (150 m) + Linear feature crossing rate	317.32	0.00	0.54	−153.66
Season + Distance to secondary road (50 m) + Distance to residence (150 m) + Linear feature crossing rate	318.85	1.53	0.25	−155.42
Step Length + Season + Distance to residence (150 m) + Linear feature crossing rate	319.82	2.50	0.15	−155.91
Season + Distance to residence (150 m) + Linear feature crossing rate	322.57	5.25	0.04	−158.28
Step length + Season + Distance to secondary road (50 m) + Linear feature crossing rate	325.74	8.42	0.01	−158.87

**Table 7:** Parameter estimates from the best-supported Cox proportional hazard model describing cougar survival in west-central Alberta, Canada.

Variable	Hazard Ratio	P	Lower Confidence	Upper Confidence	Likelihood Ratio Test
Step Length	1.11	0.262	0.92	1.32	4E−12
Linear feature crossing rate	1.22	< 0.001	1.095	1.36	
Season	13.33	< 0.001	3.10	57.44	
Distance to residence (150m)	11.36	< 0.001	3.19	40.43	
Distance to secondary road (50m)	2.83	0.023	1.15	6.92	

## Discussion

Human-caused mortality, particularly from sport harvest, often exceeds natural causes for large carnivores [9,12,32,57]. Where harvest is a primary source of mortality, males frequently experience lower survival due to sex-selective harvest [18,21,58,59]. Consistent with other regions, we found harvest was the leading cause of mortality, however, there was no statistically significant difference in survival between sexes and no evidence of male-biased mortality risk from harvest. Harvest-related mortality was low (43%) compared to other areas reporting 62–75% [17,18], though considerable regional variation exists. Some populations with lower overall harvest rates (e.g., 25%) have nevertheless found males had lower survival than females [21]. In our study, harvest accounted for a greater proportion of male mortalities (64%) than female mortalities (35%), but this difference was not statistically significant and we did not detect a sex effect in harvest mortality risk. This pattern may reflect more balanced harvest pressure between males and females in this system, although limited sample size, particularly for males, may have reduced power to detect sex-specific differences. We hypothesized that age class would influence survival, but found no support. Age-related patterns may be obscured by dispersal behaviours and their associated risks, such as increased movement rates, unfamiliarity with new environments, or other extrinsic factors [26–28]. Evidence from Washington illustrates that heavy harvest regimes (utilizing hounds) can be a primary determinant of population demographics, significantly reducing kitten survival and shifting populations toward a younger age structure [25]. Similarly, research from the Cypress Hills has shown that subadult cougars employ behavioral strategies to mitigate risk, including rapid movements at night and “leap-frog” dispersal patterns used to traverse unfamiliar landscapes, potentially obscuring age-related patterns in survival [27]. Together, these findings suggest that extrinsic factors, including harvest intensity and landscape connectivity, may be more influential drivers of cougar survival than age class alone. A larger sample size, however, may provide greater insights.

We expected environmental factors such as season, terrain, and proximity to edges to influence cougar survival by altering vulnerability to harvest. Of these, only season was retained as a significant predictor, with survival lower during the cold season. In contrast, terrain ruggedness and proximity to forest edges, variables that were expected to increase visibility or hunter access, were not significant, likely reflecting the use of hounds for hunting. Under these conditions, visual detection from edges may be less important than locating tracks.

We hypothesized that anthropogenic features related to human access and density would reduce survival, with higher mortality risk near roads, residences, and other developed areas due to increased harvest pressure [21]. Our analyses supported this hypothesis, as the top model included distance to residences, distance to secondary roads, and linear-feature crossing rate. Mortality risk increased with proximity to residences and secondary roads, indicating that cougars occupying accessible areas were more vulnerable to harvest. These findings suggest that risk is driven less by general development intensity and more by localized access networks that facilitate hunter access. Linear-feature crossing rate was also a strong predictor of mortality, consistent with hunters using these features to locate tracks before pursuit. We interpret increased anthropogenic feature use as a proxy for mechanisms such as elevated human encounter risk or hunting pressure, rather than a direct effect of the physical footprint itself on survival. However, the intensity of human use of these features was not directly measured. These results suggest that cougar mortality risk is strongly structured by landscape access, which has direct implications for how harvest exposure is distributed under current management regimes. While overall survival estimates were comparable to other harvested populations, our results highlight the importance of considering spatial patterns of access when evaluating the sustainability and distribution of human-caused mortality.

We hypothesized that the best-supported model for cougar survival would include biological, environmental, and anthropogenic features, as each represents complementary aspects of risk associated with harvest and overall mortality [21,31]. Our hypothesis was supported, as the top model included step length, season, distance to secondary roads, distance to residences, and linear feature crossing rate. Within this model, season and anthropogenic access variables were the strongest predictors of survival. Although step length was included in the top-supported model, it was only marginally stronger than the second-best model that did not include step length and was not a statistically significant predictor, suggesting that movement behaviour likely played a limited or indirect role in mortality risk. Collectively, these results indicate that cougar survival was shaped by environmental factors emphasizing the interactive effects of behaviour, season, and access on mortality risk.

Our results have direct implications for cougar management in systems where harvest is the primary source of mortality, particularly in relation to how landscape access influences exposure risk and how sex-specific harvest patterns are interpreted. We also acknowledge several limitations that may influence inference, including sample size constraints and the lack of direct measures of hunter effort and harvest intensity. In particular, uneven sample sizes across sex and age classes and relatively low numbers of mortality events may have reduced statistical power, increasing uncertainty around effect estimates and limiting our ability to detect weaker relationships. Future research should prioritize sampling more males to better understand potential inter-sex differences in survival and responses to risk factors. We acknowledge that comparisons between survival estimates and sources of mortality may be biased due to our female-dominated sample. A clearer understanding of the relative contribution of different mortality sources would also help identify where and when each sex is most vulnerable. Studies have suggested that females may experience higher natural mortality due to intraspecific aggression while defending kittens [58]. We documented three adult females killed by wolves, two of which had dependent offspring, supporting the idea that female mortality can be elevated by natural causes associated with maternal defense. Additional research is needed to further assess the risks of maternal care and its influence on female survival. Conversely, higher male mortality is often attributed to selective harvest, as hunters using hounds often select large males [60]. In our study area, management regulations are structured in ways that accentuate this bias: harvest quotas are male-biased, and females accompanied by kittens are protected from harvest. Under these conditions, combined with a general preference to harvest males, we would expect male harvest mortality to exceed that of females. However, we found that mortality risk from harvest did not differ by sex. This suggests that either (1) preferential harvest of males was minimal, meaning that although male quotas were higher, they did not result in disproportionate male harvest, or (2) the smaller number of monitored males limited our ability to detect a difference. We recommend increasing the number of monitored males to increase statistical power.

## Supporting information

S1 File Descriptive tables and figures of cougar survival in west-central Alberta (2016–2021). (DOCX)

S2 File Additional methods for variables excluded from final analyses. (DOCX)

## References

[1] Noss RF, Quigley HB, Hornocker MG, Merrill T, Paquet PC. Conservation biology and carnivore conservation in the Rocky Mountains. Conserv Biol. 1996;10:949–63.

[2] Ritchie EG, Johnson CN. Predator interactions, mesopredator release and biodiversity conservation. Ecol Lett. 2009;12(9):982–98. doi: 10.1111/j.1461-0248.2009.01347.x 19614756

[3] Ripple WJ, Estes JA, Beschta RL, Wilmers CC, Ritchie EG, Hebblewhite M, et al. Status and ecological effects of the world’s largest carnivores. Science. 2014;343(6167):1241484. doi: 10.1126/science.1241484 24408439

[4] Hebblewhite M, Whittington J. Wolves without borders: Transboundary survival of wolves in Banff National Park over three decades. Glob Ecol Conserv. 2020;24:e01293. doi: 10.1016/j.gecco.2020.e01293

[5] Miller SD, McLellan BN, Derocher AE. Conservation and management of large carnivores in North America. Int J Environ Stud. 2013;70(3):383–98. doi: 10.1080/00207233.2013.801628

[6] Teichman KJ, Cristescu B, Darimont CT. Hunting as a management tool? Cougar-human conflict is positively related to trophy hunting. BMC Ecol. 2016;16(1):44. doi: 10.1186/s12898-016-0098-4 27729084 PMC5057462

[7] Clark DA, Johnson BK, Jackson DH. Monthly and annual survival rates of cougar kittens in Oregon. Northwest Sci. 2015;89(4):393–400. doi: 10.3955/046.089.0407

[8] Treves A, Karanth U. Human-carnivore conflict and perspectives on carnivore management worldwide. Conserv Biol. 2003;17:1491–9.

[9] Boulanger J, Cattet M, Nielsen SE, Stenhouse G, Cranston J. Use of multi‐state models to explore relationships between changes in body condition, habitat and survival of grizzly bears *Ursus arctos horribilis*. Wildl Biol. 2013;19(3):274–88. doi: 10.2981/12-088

[10] Gardner CL, Pamperin NJ, Benson JF. Movement patterns and space use of maternal grizzly bears influence cub survival in interior Alaska. Ursus. 2014;25(2):121–38. doi: 10.2192/ursus-d-14-00015.1

[11] Keehner JR, Wielgus RB, Maletzke BT, Swanson ME. Effects of male targeted harvest regime on sexual segregation in mountain lion. Biol Conserv. 2015;192:42–7. doi: 10.1016/j.biocon.2015.09.005

[12] Lee DJ, Vaughan MR, Martin K. Yearling and subadult black bear survival in a hunted Virginia population. J Wildl Manage. 2005;69(4):1641–51. doi: 10.2193/0022-541X(2005)69[1641:Yasbbs]2.0.Co;2

[13] Hebblewhite M, Percy M, Serrouya R. Black bear (*Ursus americanus*) survival and demography in the Bow Valley of Banff National Park, Alberta. Biol Conserv. 2003;112(3):415–25. doi: 10.1016/s0006-3207(02)00341-5

[14] Nielsen S, Boyce MS, Stenhouse GB. Grizzly bears and forestry I. Selection of clearcuts by grizzly bears in west-central Alberta, Canada. Forest Ecol Manage. 2004;199(1):51–65. doi: 10.1016/j.foreco.2004.04.014

[15] Hiller TL, McFadden-Hiller JE, Jenkins SR, Belant JL, Tyre AJ. Demography, prey abundance, and management affect number of cougar mortalities associated with livestock conflicts. J Wildl Manage. 2015;79(6):978–88. doi: 10.1002/jwmg.913

[16] Young J, Tewes M, Haines A, Guzman G, Demaso S. Survival and mortality of cougars in the Trans-Pecos region. Southwest Nat. 2010;55(3):411–8. doi: 10.1894/MD-10.1

[17] Wolfe ML, Koons DN, Stoner DC, Terletzky P, Gese EM, Choate DM. Is anthropogenic cougar mortality compensated by changes in natural mortality in Utah? Insight from long-term studies. Biol Conserv. 2015;182:187–96. doi: 10.1016/j.biocon.2014.12.008

[18] McKinney T, Smith T, Waddell R. Rates of survival and sources of mortality of cougars in hunted populations in north-central Arizona. Southwest Nat. 2009;54(2):151–5.

[19] Schwab AC, Zandbergen PA. Vehicle-related mortality and road crossing behavior of the Florida panther. Appl Geogr. 2011;31(2):859–70. doi: 10.1016/j.apgeog.2010.10.015

[20] Logan KA, Sweanor LL. Desert puma: evolutionary ecology and conservation of an enduring carnivore. Washington, D.C., USA: Island Press; 2001.

[21] Ruth TK, Haroldson MA, Murphy KM, Buotte PC, Hornocker MG, Quigley HB. Cougar survival and source-sink structure on Greater Yellowstone’s northern range. J Wildl Manage. 2011;75(6):1381–98. doi: 10.1002/jwmg.190

[22] Knopff AA, Knopff KH, Boyce MS, St. Clair CC. Flexible habitat selection by cougars in response to anthropogenic development. Biol Conserv. 2014;178:136–45. doi: 10.1016/j.biocon.2014.07.017

[23] Smereka CA, Frame PF, Edwards MA, Frame DD, Slater OM, Derocher AE. Seasonal habitat selection of cougars *Puma concolor* by sex and reproductive state in west‐central Alberta, Canada. Wildl Biol. 2020;2020(4):1–14. doi: 10.2981/wlb.00735

[24] Dickie M, Serrouya R, McNay RS, Boutin S, du Toit J. Faster and farther: Wolf movement on linear features and implications for hunting behaviour. J Appl Ecol. 2017;54(1):253–63. doi: 10.1111/1365-2664.12732

[25] Cooley HS, Wielgus RB, Koehler GM, Robinson HS, Maletzke BT. Does hunting regulate cougar populations? A test of the compensatory mortality hypothesis. Ecology. 2009;90(10):2913–21. doi: 10.1890/08-1805.1 19886499

[26] McLellan BN, Hovey FW. Natal dispersal of grizzly bears. Can J Zool. 2001;79(5):838–44. doi: 10.1139/z01-051

[27] Morrison CD, Boyce MS, Nielsen SE. Space-use, movement and dispersal of sub-adult cougars in a geographically isolated population. PeerJ. 2015;3:e1118. doi: 10.7717/peerj.1118 26290786 PMC4540023

[28] Thorsen NH, Hansen JE, Støen O-G, Kindberg J, Zedrosser A, Frank SC. Movement and habitat selection of a large carnivore in response to human infrastructure differs by life stage. Mov Ecol. 2022;10(1):52. doi: 10.1186/s40462-022-00349-y 36447280 PMC9706841

[29] Smereka CA, Frame PF, Edwards MA, Slater OM, Frame DD, Derocher AE. Space use of cougars at the northern edge of their range. J Mammal. 2021;102(4):1042–53. doi: 10.1093/jmammal/gyab070

[30] Dellinger JA, Cristescu B, Ewanyk J, Gammons DJ, Garcelon D, Johnston P. Using mountain lion habitat selection in management. J Wildl Manage. 2019;84(2):359–71. doi: 10.1002/jwmg.21798

[31] Smith JA, Wang Y, Wilmers CC. Top carnivores increase their kill rates on prey as a response to human-induced fear. Proc Biol Sci. 2015;282(1802):20142711. doi: 10.1098/rspb.2014.2711 25608884 PMC4344154

[32] Holmes BR, Laundré JW. Use of open, edge and forest areas by pumas *Puma concolor* in winter: are pumas foraging optimally?. Wildlife Biol. 2006;12(2):201–9. doi: 10.2981/0909-6396(2006)12[201:uooeaf]2.0.co;2

[33] Elbroch LM, Lendrum PE, Alexander P, Quigley H. Cougar den site selection in the Southern Yellowstone Ecosystem. Mamm Res. 2015;60(2):89–96. doi: 10.1007/s13364-015-0212-6

[34] Benson JF, Lotz MA, Jansen D. Natal den selection by Florida panthers. J Wildl Manage. 2008;72(2):405–10. doi: 10.2193/2007-264

[35] Morrison CD, Boyce MS, Nielsen SE, Bacon MM. Habitat selection of a re-colonized cougar population in response to seasonal fluctuations of human activity. J Wildl Manage. 2014;78(8):1394–403. doi: 10.1002/jwmg.799

[36] Kertson BN, Spencer RD, Marzluff JM, Hepinstall-Cymerman J, Grue CE. Cougar space use and movements in the wildland–urban landscape of western Washington. Ecological Applications. 2011;21(8):2866–81. doi: 10.1890/11-0947.1

[37] Peebles KA, Wielgus RB, Maletzke BT, Swanson ME. Effects of remedial sport hunting on cougar complaints and livestock depredations. PLoS One. 2013;8(11):e79713. doi: 10.1371/journal.pone.0079713 24260291 PMC3834330

[38] Government of Alberta. Management Plan for Cougars in Alberta. Wildlife Management Planning Series Number 8 Alberta Environment and Sustainable Resource Development Wildlife Branch Edmonton, Alberta; 2012.

[39] Downing DJ, Pettapiece WW. Natural regions and subregions of Alberta. Natural Resource Committee. Government of Alberta. 2006.

[40] Knopff A, Knopff K, Kortello A, Boyce MS. Cougar kill rate and prey composition in a multiprey system. J Wildl Manage. 2010;74(7):1435–47. doi: 10.2193/2009-314

[41] Littlefair CJ, Derocher AE, Frame PF, Edwards MA, Frame DD, Slater OM. Chemical immobilization effects on cougar (*Felis concolor*) movement. J Wildl Dis. 2024;60(4):985–90. 10.7589/JWD-D-23-00191 39093818

[42] Girard TL, Bork EW, Nielsen SE, Alexander MJ. Seasonal variation in habitat selection by free-ranging feral horses within Alberta’s forest reserve. Rangeland Ecol Manage. 2013;66(4):428–37. doi: 10.2111/rem-d-12-00081.1

[43] Zamar D, McNeney B, Graham J. elrm: Software implementing exact-like inference for logistic regression models. J Stat Soft. 2007;21(3). doi: 10.18637/jss.v021.i03

[44] Johnson CJ, Boyce MS, Schwartz CC, Haroldson MA. Modeling survival: Application of the Andersen–Gill model to Yellowstone grizzly bears. J Wildl Manage. 2004;68(4):966–78. doi: 10.2193/0022-541x(2004)068[0966:Msaota]2.0.Co;2

[45] Therneau T. _A package for survival analysis in R_. R package version 3.2-13. 2021.

[46] Alberta Go. 2015 Land Cover of Canada 2026. Available from: https://open.canada.ca/data/en/dataset/4e615eae-b90c-420b-adee-2ca35896caf6/resource/7b2bfd6a-da66-454b-8841-934c7661e0f8

[47] Boulanger J, Stenhouse GB. The impact of roads on the demography of grizzly bears in Alberta. PLoS One. 2014;9(12):e115535. doi: 10.1371/journal.pone.0115535 25532035 PMC4274100

[48] Nielsen S, Cranston J, Stenhouse G. Identification of priority areas for grizzly bear conservation and recovery in Alberta, Canada. J Conserv Plan. 2009;5:38–60.

[49] Banfield JE, Ciuti S, Nielsen CC, Boyce MS. Cougar roadside habitat selection: Incorporating topography and traffic. Global Ecol Conserv. 2020;23:e01186. doi: 10.1016/j.gecco.2020.e01186

[50] Burnham KP, Anderson DR, Huyvaert KP. AIC model selection and multimodel inference in behavioral ecology: Some background, observations, and comparisons. Behav Ecol Sociobiol. 2010;65(1):23–35. doi: 10.1007/s00265-010-1029-6

[51] Natural Resources Canada. Pipeline Infrastructure. 2017.

[52] Natural Resources Canada. Powerlines. 2020.

[53] Natural Resources Canada. Access. 2018.

[54] Calenge C. The package “adehabitat” for the R software: A tool for the analysis of space and habitat use by animals. Ecol Modell. 2006;197(3–4):516–9. doi: 10.1016/j.ecolmodel.2006.03.017

[55] Freckleton RP. Dealing with collinearity in behavioural and ecological data: Model averaging and the problems of measurement error. Behav Ecol Sociobiol. 2010;65(1):91–101. doi: 10.1007/s00265-010-1045-6

[56] Schober P, Boer C, Schwarte LA. Correlation coefficients: Appropriate use and interpretation. Anesth Analg. 2018;126(5):1763–8. doi: 10.1213/ANE.0000000000002864 29481436

[57] Chakrabarti S, O’Neil ST, Erb J, Humpal C, Bump JK. Recent trends in survival and mortality of wolves in Minnesota, United States. Front Ecol Evol. 2022;10. doi: 10.3389/fevo.2022.826358

[58] Robinson HS, Wielgus RB, Cooley HS, Cooley SW. Sink populations in carnivore management: Cougar demography and immigration in a hunted population. Ecol Appl. 2008;18(4):1028–37. doi: 10.1890/07-0352.1 18536260

[59] Czetwertynski SM, Boyce MS, Schmiegelow FK. Effects of hunting on demographic parameters of American black bears. Ursus. 2007;18(1):1–18. doi: 10.2192/1537-6176(2007)18[1:eohodp]2.0.co;2

[60] Martorello DA, Beausoleil RA. Characteristics of cougar harvest with and without the use of dogs. 2003: Proceedings of the Seventh Mountain Lion Workshop. Wyoming Game and Fish Department, Lander, Wyoming, USA. p. 129–35.

