# Supplementary material for: Biological and environmental correlates of cougar (Puma concolor) survival in west central Alberta
Source: PLoS One. 2026 Oct 28;21(10):e0357879. doi: 10.1371/journal.pone.0357879 (PMC13716201; doi:10.1371/journal.pone.0357879)
Supplement: Supplementary file 1 [file pone.0357879.s001.docx]

**S1 File. Descriptive tables and figures of cougar survival in west-central Alberta (2016-2021).**

**Figure S1.** Survival estimates for female cougars with and without dependents within west central Alberta, Canada, between 2016 and 2021. A total of 56 females were monitored during the study including 18 with offspring.

**Table S1.** Number of cougars captured, collared, and monitored in west-central Alberta, Canada, between 2016 and 2021, with descriptive statistics of monitoring duration by sex.

|  | **Individuals** | **Months Monitored** | **Mean** | **Median** | **Standard Deviation** |
| --- | --- | --- | --- | --- | --- |
| Female | 56 | 812.2 | 14.5 | 10.9 | 12.1 |
| Male | 24 | 269.8 | 11.2 | 10.3 | 7.9 |
| Total | 80 | 1082.0 | 13.5 | 10.7 | 11.0 |
|  |  |  |  |  |  |

**Table S2.** Kaplan–Meier survival estimates and 95% confidence intervals for collared cougars in west-central Alberta, Canada (2016 – 2021), calculated annually and for warm (April – October) and cold (November – March) seasons by sex and age class.

|  | | **N** |  | **Annual** | |  | **Warm Season** | |  | **Cold Season** | |
| --- | --- | --- | --- | --- | --- | --- | --- | --- | --- | --- | --- |
| Sex / Age Class | |  |  | Survival | CI |  | Survival | CI |  | Survival | CI |
| Male | |  |  |  |  |  |  |  |  |  |  |
|  | Subadult | 7 |  | 0.857 | 0.633 - 1 |  | 1 | 1 - 1 |  | 1 | 1 - 1 |
|  | Adult | 23 |  | 0.586 | 0.406 - 0.845 |  | 1 | 1 - 1 |  | 0.827 | 0.685 - 0.999 |
|  | |  |  |  |  |  |  |  |  |  |  |
| Female | |  |  |  |  |  |  |  |  |  |  |
|  | Subadult | 14 |  | 0.857 | 0.692 - 1 |  | 1 | 1 – 1 |  | 0.929 | 0.803 - 1 |
|  | Adult | 53 |  | 0.628 | 0.512 - 0.770 |  | 0.937 | 0.879 - 0.999 |  | 0.726 | 0.578 - 0.913 |

**Table S3.** Variables excluded from final survival analyses due to non-significance in preliminary Cox proportional hazards models (AIC_c_).

| **Classification** | **AIC_c_** | **Delta AIC_c_** | **AICc Wt.** | **LL** | **K** |
| --- | --- | --- | --- | --- | --- |
| Forest Pooled | 373.38 | 0.00 | 0.89 | -179.45 | 6 |
| Forest Separate | 377.63 | 4.25 | 0.11 | -178.56 | 8 |
|  |  |  |  |  |  |
| Forest Edge 50 m | 370.92 | 0.00 | 0.55 | -184.41 | 1 |
| Forest Edge 100 m | 371.33 | 0.40 | 0.45 | -184.61 | 1 |
|  |  |  |  |  |  |
| Water 100 m | 369.45 | 0.00 | 0.56 | -183.67 | 1 |
| Water 50 m | 369.96 | 0.51 | 0.44 | -183.93 | 1 |
|  |  |  |  |  |  |
| Industrial 50 m | 371.12 | 0.00 | 0.36 | -184.51 | 1 |
| Industrial 150 m | 371.18 | 0.06 | 0.35 | -184.54 | 1 |
| Industrial 250 m | 371.56 | 0.44 | 0.29 | -184.73 | 1 |
|  |  |  |  |  |  |
| Primary Road 250 m | 370.85 | 0.00 | 0.40 | -184.37 | 1 |
| Primary Road 150 m | 371.17 | 0.33 | 0.34 | -184.54 | 1 |
| Primary Road 50 m | 371.66 | 0.82 | 0.26 | -184.78 | 1 |

**Table S4.** Variables excluded during preliminary analyses due to lack of significance in univariate Cox proportional hazard models.

| **Category** | **Variable** | **Hazard Ratio** | **P** | **Likelihood Ratio Test** |
| --- | --- | --- | --- | --- |
| Biological | Sex | 1.44 | 0.27 | 0.3 |
|  | Age Class Kitten | 1.71 | 0.31 | 0.6 |
|  | Subadult | 0.96 | 0.94 |  |
| Environmental | Landcover | - | 0.98 - 1.0 | 0.1 |
|  | Elevation | 1 | 0.98 | 1 |
|  | Slope | 0.93 | 0.29 | 0.2 |
|  | VRM | 2.43E-202 | 0.15 | 0.08 |
|  | Time Since Snow | 0.99 | 0.19 | 0.1 |
|  | Water Distance (100m) | 0.31 | 0.21 | 0.1 |
|  | Edge Distance (50m) | 1.35 | 0.39 | 0.4 |
| Anthropogenic | Primary Road Density | 0.90 | 1.00 | 0.5 |
|  | Secondary Road Density | 1 | 0.38 | 0.4 |
|  | Industrial Density | 2.11E-07 | 1.00 | 0.3 |
|  | Primary Road Distance (250m) | 3.42 | 0.29 | 0.4 |
|  | Industrial Distance (50m) | - | 1.00 | 0.5 |
|  | Primary Road Crossing Rate | 1.689E-07 | 0.56 | 0.5 |

- the model failed to produce a stable hazard ratio estimate due to lack of variation in the variable.

**Table S5.** Minimum, median, and maximum values of covariates used in cougar survival and hazard analyses in west-central Alberta, Canada, from 2016 to 2021.

| Variable | Minimum | Median | Maximum |
| --- | --- | --- | --- |
| Step length | 0 | 0.29 | 16.59 |
| Linear Feature Crossing Rate | 0 | 1.89 | 16.23 |
| Secondary Road Crossing Rate | 0 | 0.21 | 2.15 |
| Primary Road Crossing Rate | 0 | 0 | 0.05 |
| VRM | 1.00x 10^-7^ | 1.56 x 10^-4^ | 0.026 |
| Slope | 0.068 | 2.46 | 44.25 |
| Elevation | 556.20 | 928.20 | 2275.00 |
| Time Since Snow (Days) | 0 | 13 | 200 |
| Water Distance (100m) | <0.01 | <0.01 | 1 |
| Edge Distance (50m) | <0.01 | 0.64 | 1 |
| Residential Density | 0 | 0 | 57 |
| Industrial Density | 0 | 0 | 7 |
| Secondary Road Density | 0 | 0 | 2442 |
| Primary Road Density | 0 | 0 | 1172 |
| Secondary Road Distance (50m) | <0.01 | <0.01 | 1 |
| Residence Distance (150m) | <0.01 | <0.01 | 1 |
| Primary Road Distance (250m) | <0.01 | <0.01 | 1 |
| Industrial Distance (50m) | <0.01 | <0.01 | 1 |

**Table S6** Pearson correlation coefficients and variance inflation factors (VIF) for anthropogenic covariates considered in cougar mortality risk models.

| Variable | 1 | 2 | 3 | 4 | 5 | VIF |
| --- | --- | --- | --- | --- | --- | --- |
| 1. Residential Density | — |  |  |  |  | 1.58 |
| 2. Residential Distance (150 m) | 0.6 | — |  |  |  | 1.58 |
| 3. Secondary Road Distance (50 m) | 0.01 | 0.08 | — |  |  | 1.04 |
| 4. Secondary Road Crossing Rate | 0.05 | 0.03 | 0.16 | — |  | 1.89 |
| 5. Linear Feature Crossing Rate | -0.02 | -0.01 | 0.04 | 0.67 | — | 1.84 |
