# Supplementary material for: Biological and environmental correlates of cougar (Puma concolor) survival in west central Alberta
Source: PLoS One. 2026 Oct 28;21(10):e0357879. doi: 10.1371/journal.pone.0357879 (PMC13716201; doi:10.1371/journal.pone.0357879)
Supplement: Supplementary file 2 [file pone.0357879.s002.docx]

**S2 File. Additional Methods for Excluded Variables.**

Methods used for variables dropped during preliminary analysis

Landcover classifications at 30 m resolution were open area, forested area, wetland, agriculture land, edge area, and water [58-60]. Forested areas were classified as coniferous, broadleaf, and mixed forests. Landcover was modeled as an annual hazard with forest types separate, and pooled (i.e., Forest Pooled). The model with the lower AIC_c_ was then used in all subsequent time frames investigated. Edge area was classified as any forest cell that bordered any other cell type (e.g., open area, wetland) and mapped by buffering 25 m into the forest cell and adjacent cell and removing this area from the original classification. Open area had no vegetation or vegetation ≤1 m tall and consisted of non-vegetated land, grassland, and herbaceous land [32, 58]. Forest type layers were acquired from Canadian Forest Service Earth Observation for Sustainable Development of Forest from 2015 with 30 m pixel resolution generated using Landsat data [59].

Within ArcGIS a digital elevation model (25m resolution) was used to determine elevation, terrain ruggedness and slope [61]. From each GPS point a 300 m radius was created and within these radii the mean elevation (m) and terrain ruggedness (vector ruggedness measure, VRM) was determined [9, 62]. Slope (%) was determined using the *Slope* tool in ArcGIS, calculated as the maximum change in elevation between the cell and its neighboring cells. Time since the last snowfall was determined as a track detection metric for hunters. Weather data was acquired from nine nearby weathers stations (i.e., Breton Plots, Fort Assiniboine, Goose Mountain Lookout, James River Ranger Station, Little Paddle Headwaters, Mayerthorpe, Nordegg, Rocky Mountain House, and Violet Grove) from the Alberta Climate Information Services [63]. Each location was assigned to the nearest weather station and the time difference between the location and the most recent snowfall was determined.

Crossing rates of primary roads were examined to calculate associated mortality risk and consisted of all paved roads. Roads were mapped in ArcGIS (version 10.7.1, Environmental Systems Research Institute, Redlands, CA) and the individual crossing rate was determined as crossings/steps.

Density from cougar locations were determined for industrial sites and roads (primary and secondary separately). From each collar point within a 300 m radius road density (km of road) and a 1 km radius building density were determined. Density of roads, and industrial buildings (annually and seasonally) were then used as covariates [51].

Euclidean distances to forest edge, water, primary roads, and industrial buildings were determined for each cougar location using the Near tool in ArcGIS. Closer locations were expected to have more of an effect than those further away so we created exponential decay layers (e^(-d/a)^), where *d* was the distance to the feature and *a* was the buffer value [23, 52]. To reduce model complexity we used a subset of variable distances used by Smereka, Frame (23), we created layers with buffer radii of 50 m and 100 m for forest edge and water bodies. For primary roads and industrial buildings, exponential decay layers with buffer radii of 50 m, 150 m, and 250 m were used. A large range of buffer layers were examined to account for cougars response to anthropogenic areas as well as human access to these areas [53]. Buffer layers were created, and Euclidean distances were measured using the Buffer and Near tool in ArcGIS. Each features buffer layer was ranked using Cox proportional hazard models as an annual hazard and the lowest AIC_c_ for each layer was then used in each seasons analysis [22].
